# Pre‐ and Post‐Interventional Hemodynamic Characterization of the Femoropopliteal Artery Using Vector Flow Imaging in Peripheral Arterial Occlusive Disease

**DOI:** 10.1002/jum.70176

**Published:** 2026-01-05

**Authors:** Jan Lukas Prüser, Gisela Winkelbauer, Wulf Dieker, Abdulkader Adler, Klaus Amendt, Martin Sigl

**Affiliations:** ^1^ Department of Medicine VI, Interdisciplinary Vascular Center, University Medical Center Mannheim, Medical Faculty Mannheim Heidelberg University Mannheim Germany

**Keywords:** angioplasty, Doppler ultrasound, femoropopliteal artery, revascularization, vector flow imaging, wall shear stress

## Abstract

**Objectives:**

The femoropopliteal (FP) artery is the most frequently revascularized segment in peripheral artery disease (PAD), followed by the iliac segment. Wall shear stress (WSS) is a key local factor implicated in both atherosclerotic plaque formation and restenosis after angioplasty. However, WSS is not routinely assessed in peri‐interventional clinical practice. This exploratory study aimed to assess the feasibility and potential utility of measuring WSS in the FP artery segment using ultrasound.

**Methods:**

In this prospective, single‐center study, we included patients with symptomatic PAD and evaluated their hemodynamic parameters before and after iliac or FP revascularization. In addition to standard ultrasound examinations—including B‐mode, color Doppler, and pulse‐wave (PW) Doppler—we performed sonographic vector flow imaging (VFI) and assessed volume flow and the WSS‐derived oscillatory shear index (OSI) at 3 predefined segments along the FP axis.

**Results:**

Following iliac or FP revascularization, PW‐derived median volume flow increased significantly at all 3 FP sites: in the common femoral artery (CFA) from 211 to 270 mL/minute (*p* < .01), in the superficial femoral artery (SFA) from 104 to 138 mL/minute (*p* < .01), and in the popliteal artery (PA) from 37 to 73 mL/minute (*p* < .001). Median WSS values also increased significantly: in the CFA from 0.69 to 0.93 Pa (*p* < .001), in the SFA from 0.78 to 1.04 Pa (*p* < .05), and in the PA from 0.78 to 0.91 Pa (*p* < .001). By contrast, OSI values showed no significant changes (range 0.0–0.12, all *p* > .3).

**Conclusions:**

Iliac or FP revascularization procedures result in measurable hemodynamic changes in FP blood flow and vessel wall interaction, which can be readily assessed using peri‐interventional ultrasound. The clinical relevance of increased WSS along the FP axis warrants further investigation.

**Abbreviations**
CFA: common femoral artery
FP: femoropopliteal
NO: nitric oxide
OSI: oscillatory shear index
PA: popliteal artery
PAD: peripheral artery disease
PRF: pulse repetition frequency
PW: pulse‐wave
ROI: region of interest
SD: standard deviation
SFA: superficial femoral artery
VFI: vector flow imaging
WSS: wall shear stress

Peripheral artery disease (PAD) is a common manifestation of atherosclerosis, currently affecting more than 200 million people worldwide, with its prevalence continuing to rise.^1^ The number of peripheral vascular interventions has also increased significantly, with the femoropopliteal (FP) artery being the most frequently revascularized segment, followed by the iliac artery.^2^ Nevertheless, detailed hemodynamic evaluations along the FP axis, particularly in relation to revascularization procedures, remain limited.

The distribution of atherosclerotic lesions and the segmental involvement of arteries in PAD are not yet fully understood. Systemic risk factors such as smoking, hypertension, hyperlipidemia, and diabetes mellitus only partially explain the patterns observed in lower limb atherosclerosis.^3^ Increasing attention has turned to local hemodynamic forces, which may help elucidate the site‐specific nature of plaque development–frequently observed at arterial branches, bends, and bifurcations.^4^, ^5^ Among these forces, wall shear stress (WSS) has emerged as a particularly important factor in the pathogenesis of atherosclerosis and restenosis following peripheral interventions. Restenosis, especially after FP interventions, remains a major clinical concern, with reported rates of up to 70%.^6^ Accumulating evidence suggests that low WSS is associated with neointimal hyperplasia and in‐stent restenosis,^7^ highlighting its clinical importance. However, most studies on WSS have focused on large central arteries, such as the aorta and carotid arteries. In contrast, peripheral arteries—such as the FP artery—tend to be smaller in diameter, more calcified, and exhibit lower or more turbulent blood flow, posing challenges for high‐resolution flow imaging. Standard non‐invasive imaging techniques, such as magnetic resonance angiography, often lack the spatial resolution required for accurate assessment.^8^ While computed tomography offers higher spatial resolution, it involves contrast exposure and radiation, making it unsuitable for repeated or routine evaluations. One of the earliest ultrasound‐based WSS mapping studies manually assessed peak flow velocities and vessel diameters across 5 stented segments of the superficial femoral artery, finding that WSS progressively declined along the stented segment.^9^ New ultrasonographic techniques have since emerged. For instance, Wang et al performed WSS mapping of the femoral bifurcation in healthy volunteers using ultrafast vector Doppler ultrasound.^10^ More recently, high‐frame‐rate vector flow imaging (VFI) has demonstrated feasibility in assessing the FP artery in patients with PAD.^11^ Compared to standard color‐ and pulsed‐wave (PW) Doppler sonography, the conventional method for hemodynamic evaluation in PAD,^12^ VFI offers angle‐independent imaging with superior temporal and spatial resolution, enabling the assessment of WSS and the oscillatory shear index (OSI).^13^

The objective of this study was to analyze hemodynamic parameters along the FP axis in patients with symptomatic PAD before and after iliac or FP vascular interventions. We utilized color and PW Doppler ultrasound, alongside VFI, to evaluate WSS and OSI. Our goal was to enhance understanding of how revascularization influences vascular hemodynamics, potentially informing long‐term outcome prediction and treatment optimization strategies.

## Patients and Methods

### Study Design and Patient Selection

We conducted a single‐center prospective interventional study involving patients with symptomatic PAD referred to the University Vascular Center in Mannheim, Germany, for percutaneous revascularization of iliac or FP steno‐occlusive atherosclerotic disease. To ensure consistent cardiac cycle durations and minimize the impact of heart rate variability on femoral and popliteal artery volume flow, only patients with normofrequent sinus rhythm were included. Based on prior experience,^11^ only those with adequate sonographic access at predefined measurement sites were enrolled.

Image quality was classified as adequate if the ultrasound image allowed clear separation of the vessel lumen from the vessel wall, the vector representation of blood flow did not show underfilling of the vessel lumen or flow artifacts in the surrounding tissue, and the slow motion of the VFI showed at least a PW Doppler (mono‐, bi‐, or triphasic) flow profile.

### Ultrasound Imaging and Data Analysis

Sonographic recordings of all study participants were performed after at least 10 minutes of rest in the supine position. Ultrasound examinations were performed using a Resona 7 ultrasound scanner (Mindray, Shenzhen, China) equipped with the V Flow 3.0 software package (a high‐frame rate VFI tool^14^), and a linear standard transducer (L9‐U3). Standard assessments included B‐mode, color Doppler, spectral Doppler, and longitudinal VFI. Quantitative hemodynamic evaluation was performed at 3 locations: the common femoral artery (CFA), proximal superficial femoral artery (SFA; 1–3 cm distal to the femoral bifurcation), and the popliteal artery (PA; at the level of the knee joint).

#### B‐Mode Imaging

Predefined vessel segments were displayed in a longitudinal view, with alignment as parallel as possible to the transducer surface. Vessel depth was measured as the distance from the anterior wall of the artery to the skin surface. Vessel diameter was measured as the inner‐to‐inner luminal width to provide precise data for volume flow calculations.

#### Color Flow Doppler

Color Doppler imaging of predefined vessel sections was performed in a longitudinal view with a frame rate of 24 Hz and a fixed pulse repetition frequency (PRF, velocity range ± 26 cm/second). After achieving the correct angle of insonation, the color Doppler gain was adjusted, and a recording of at least 1.5 seconds in length was obtained.

#### Pulsed‐Wave Doppler

PW Doppler analysis was performed with a maximum angle correction of 60° and a sample volume set at two‐thirds of the internal vessel diameter in the longitudinal view. The angle correction was set to be within the direction of flow parallel to the vessel wall. Peak systolic, end‐diastolic, and mean velocities were recorded. Time‐averaged maximal velocity and volume flow were automatically calculated based on measurements from 3 cardiac cycles.

#### Vector Flow Imaging

VFI was used to generate 2‐dimensional velocity vector maps. Backscattered echoes were recombined offline to reconstruct vector velocities and produce a region of interest (ROI) image using plane‐wave acquisition and multi‐angle Doppler analysis.^15^ The ROI has a standard preset size of 24 mm × 20 mm, with the longer sides oriented parallel to the transducer surface. By recording for at least 1.5 seconds, a minimum of 1 cardiac cycle was captured. Assuming a circular vessel cross‐section, volume flow was calculated by integrating all velocity components across the inner arterial diameter. Volume flow calculations were automatically processed by the Resona 7 ultrasound system. Additionally, 6 WSS measurements (3 each on the near and far vessel walls) were taken by aligning a correction line perpendicular to the vessel wall and overlapping the reference centerline with the inner arterial wall, providing both maximum and mean WSS values for each point during 1 cardiac cycle. WSS calculations were automatically processed by the Resona 7 ultrasound system using the equation WSS = μ × (∂v/∂r)|_wall, where μ is the blood viscosity, v is the blood velocity profile, and r is the radial distance from the vessel center to the wall. Blood viscosity was set to 3.5 mPa s.

To quantify directional changes of the WSS vector at the vessel wall over a cardiac cycle, the oscillatory shear index was calculated by using the following equation:

𝑂𝑆𝐼=1/2×(1–𝐴𝑊𝑆𝑆𝑉/𝐴𝑊𝑆𝑆)

Where AWSSV is the magnitude of the time‐averaged WSS vector and AWSS is the time‐averaged WSS magnitude.

### Revascularization

Indications for percutaneous therapy were based on current guidelines, technical strategies, and antithrombotic therapy recommendations. Accordingly, both uncoated and coated balloons and stents were used following antegrade, retrograde, and crossover femoral approaches. Final angiography showed no significant residual stenoses of the iliac and FP arteries. No atherectomy devices or occlusion systems were used at the puncture sites.

The ankle‐brachial index was measured before and after the vascular intervention using a continuous‐wave Doppler probe. It was calculated by dividing the highest systolic blood pressure measured at the ankle by the highest systolic blood pressure measured at the brachial artery according to recent guidelines.^12^

### Ethics

This study was approved by the Medical Ethics Commission II of the Faculty of Medicine, University of Heidelberg, Mannheim, Germany (approval code 2022‐549). It was conducted in accordance with the principles of the 1964 Declaration of Helsinki and its subsequent amendments. Data protection complied with the European Union Data Protection Directive. Written informed consent was obtained from all patients.

### Statistics

The sample size was calculated prior to analysis. The primary outcomes were differences in hemodynamic sonographic parameters “pre‐ vs. post‐intervention.” Assuming a medium effect (Cohen's D = 0.6), a sample size of 24 was required to detect statistically significant differences (alpha = 0.05, power = 0.8, 2‐sided paired *t*‐test). Categorical data are presented as counts and percentages. Continuous variables are reported as means ± standard deviations (SD) or as medians with interquartile ranges, depending on distribution (assessed by the Shapiro–Wilk test). Paired continuous variables (eg, pre‐ versus post‐intervention) were compared using either a paired *t*‐test or the Wilcoxon test. A *p*‐value <.05 was considered statistically significant. Data analysis, graph generation, and statistics were performed using Excel and PowerPoint (Microsoft Corporation, USA), DATAtab (DATAtab, Austria), and SPSS Statistics 23 (SPSS Inc., USA).

## Results

**Figure 1:**
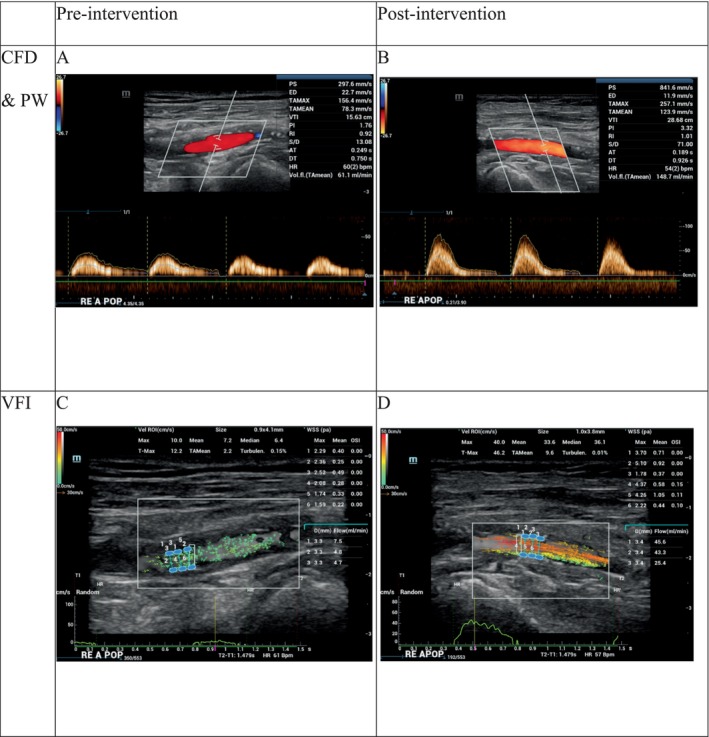
The pictures represent longitudinal ultrasound images of the popliteal artery before and after vascular intervention. Images **A** and **B** display representative measurements of volume flow based on time‐averaged mean velocity (Vol.fl. TAmean), as assessed by PW‐Doppler‐ultrasound. Images **C** and **D** illustrate measurements of volume flow, maximum and mean wall shear stress, and the oscillatory shear index using vector flow imaging. The blue ellipses indicate the areas where WSS measurements were performed, with correction lines (blue) oriented perpendicular to the vessel wall. An average of 6 measurements was used, 3 from the vessel wall near the transducer and 3 from the vessel wall farther from the transducer.

Forty patients with PAD were screened between January 2024 and April 2025. Five patients were excluded due to irregular sinus rhythm or refusal to participate. Ultimately, 35 patients were eligible. Of these, 11 did not meet predefined image quality criteria. Thus, paired datasets from 24 patients with adequate vector flow imaging were included in the analysis. Examples of pre‐ and post‐intervention ultrasound images are shown in Figure 1.

The mean patient age was 70.2 years. The most common comorbidities were hypertension (75%), diabetes mellitus (54%), and chronic renal insufficiency (54%). Angioplasty was performed in 13 patients for intermittent claudication (Rutherford category 2–3) despite adequate conservative therapy, and in 11 patients for critical limb ischemia (Rutherford category 4 or 5). Patient characteristics are summarized in Table 1.

**Table 1:** Baseline Characteristics of the Study Population with Peripheral Arterial Disease

Patients	24 (100)
Sex	
Male	13 (54)
Female	11 (46)
Age, years	70.2 ± 11.1
Rutherford clinical category	
2 (moderate claudication)	10 (42)
3 (severe claudication)	3 (13)
4 (ischemic rest pain)	2 (8)
5 (minor tissue loss)	9 (38)
Ankle‐brachial‐index	
Prior to revascularisation	0.60 ± 0.29
After revascularisation	0.88 ± 0.21
Side of intervention	
Left	10 (42)
Right	14 (58)
Target lesion localization	
Iliac	9 (38)
Femoropopliteal	14 (58)
Both	1 (4)
Tibial runoff	
1‐vessel	8 (33)
2‐vessel	4 (17)
3‐vessel	12 (50)
Prior percutaneous or open‐surgical revascularisation	8 (33)
Hyperlipidaemia	21 (88)
Hypertension	18 (75)
Ex‐ or active nicotine consumption	18 (75)
Chronic renal insufficiency (GFR <60 mL/minute)	13 (54)
Diabetes mellitus	13 (54)

Measures are given as mean ± standard deviation or number (percentage). GFR, glomerular filtration rate.

On the day following revascularization, ABI values were significantly higher than the initial values (0.88 ± 0.21 versus 0.60 ± 0.29; *p* = .002).

Both VFI‐ and PW‐derived volume flows increased at all 3 FP sites. The following volume flow and WSS data are presented as median (interquartile range). The PW‐derived median volume flow before vascular intervention in the CFA, SFA, and PA was 211 (151–237), 104 (53–140), and 37 (27–46) mL/minute, respectively. After vascular intervention, the median PW‐derived volume flows in the CFA, SFA, and PA increased to 270 (216–340), 138 (91–187), and 73 (45–87) mL/minute, respectively. The differences were statistically significant (*p* < .01 for CFA and SFA; *p* < .001 for PA).

**Figure 2:**
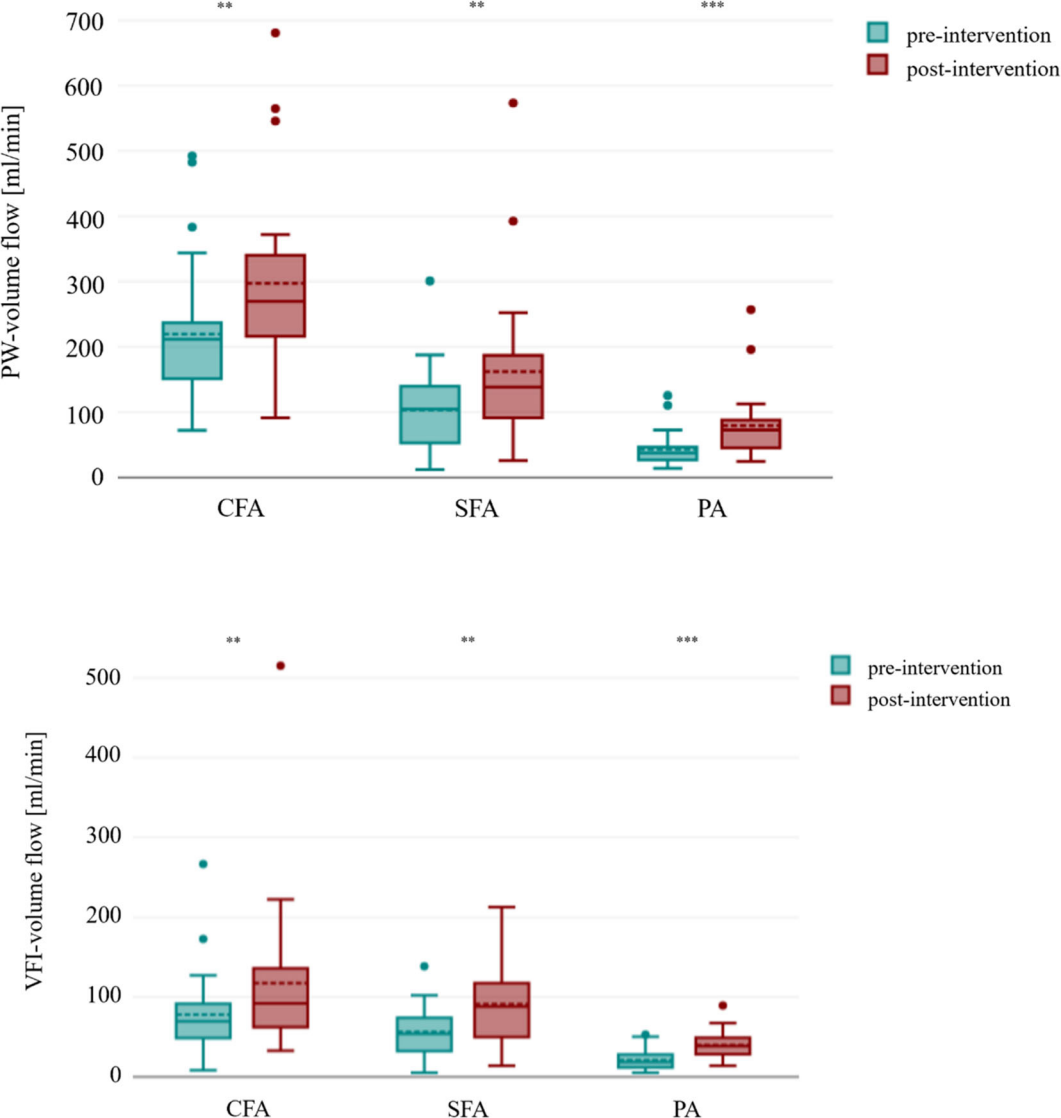
Grouped boxplot of pre‐ and post‐interventional PW‐derived (**A**) VFI‐derived volume (**B**) volume flow estimates at 3 femoropopliteal sites. Both VFI‐ and PW‐derived volume flow increased significantly at all 3 sites (**p < .01; ***p < .001). CFA, common femoral artery; PA, popliteal artery; PW, pulsed‐wave (Doppler); SFA, superficial femoral artery (proximal segment); VFI, vector flow imaging.

**Figure 3:**
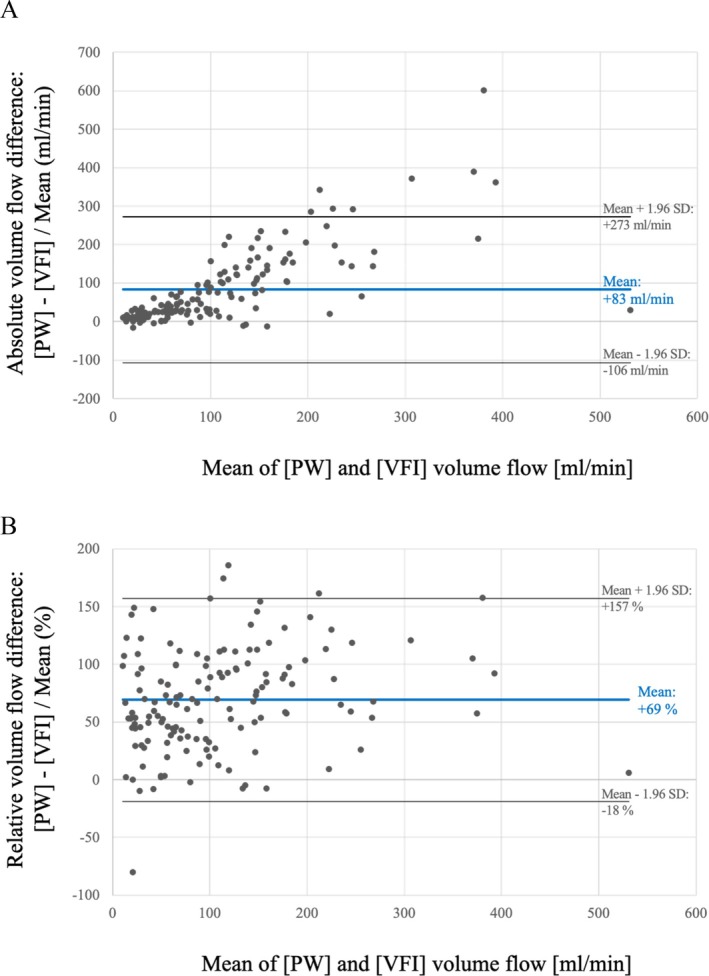
Bland–Altman plots for illustrating the differences in the estimated PW‐ and VFI‐derived volume flow, expressed as absolute values (**A**) and percentages of the values (**B**) on the y axis.

The VFI‐derived median volume flow prior to vascular intervention in the CFA, SFA, and PA was 69 (49–92), 54 (32–74), and 19 (12–28) mL/minute, respectively. The Post‐interventional VFI‐derived volume flow increased to 92 (62–136), 88 (50–117), and 39 (28–49) mL/minute, respectively. Similarly, all differences were statistically significant (*p* < .01 for CFA and SFA; *p* < .001 for PA). The grouped boxplot charts are shown in Figure 2. The PW‐derived values were significantly higher than the VFI‐derived values at all 3 sites (*p* ≤ .01 for every single site before and after vascular intervention). A Bland–Altman analysis demonstrated a systematic bias of +83.5 mL/minute (+69%) on average, with PW‐derived volume flow yielding higher values than VFI‐derived volume flow (Figure 3, A and B). This difference was statistically significant (*p* < .001; Wilcoxon test). The dispersion of differences increased with higher mean values.

**Figure 4:**
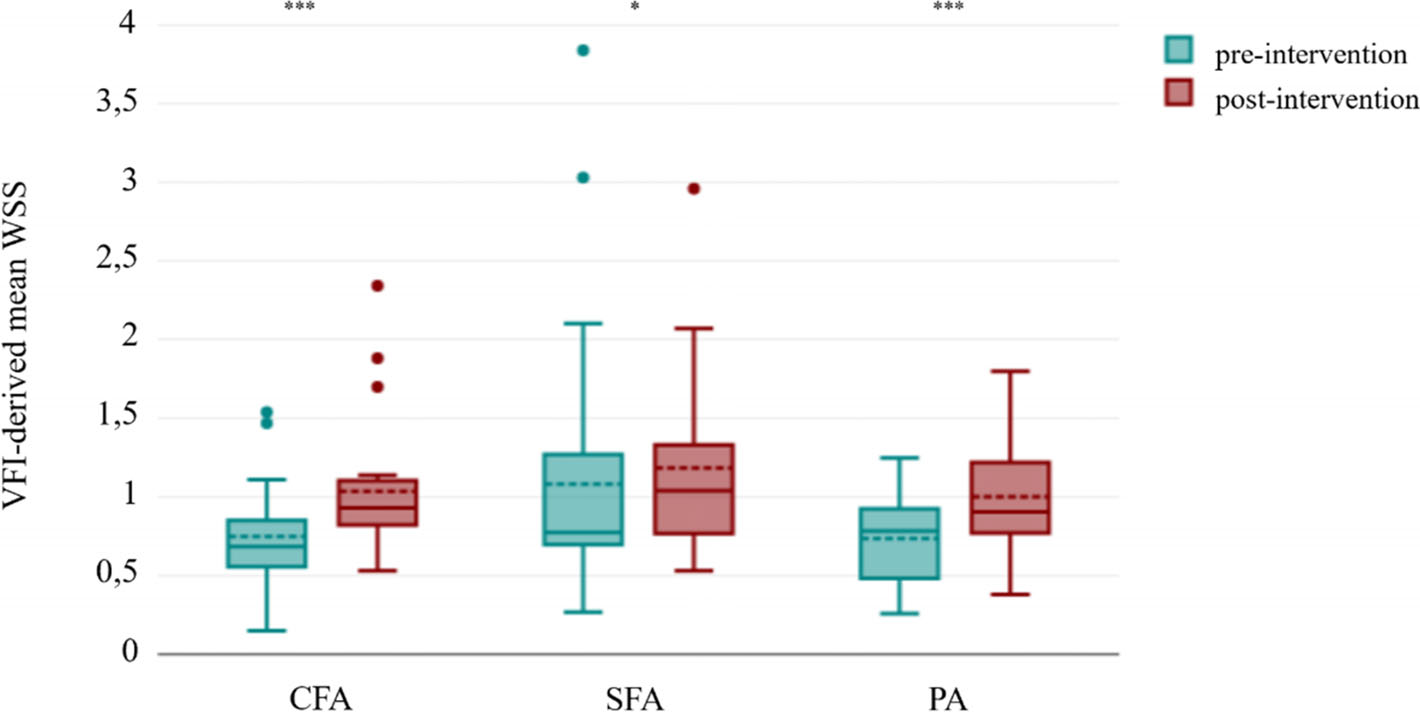
Grouped boxplot of pre‐ and post‐interventional mean wall shear stress (WSS) at 3 femoropopliteal sites. Mean WSS was calculated as the average of 6 measurements (3 each on the transducer near and far vessel wall). The mean WSS values were significantly higher at all femoropopliteal sites after revascularization (***p < .001; *p < .05). CFA, common femoral artery; PA, popliteal artery; SFA, superficial femoral artery (proximal segment); VFI, vector flow imaging; WSS, wall shear stress. Solid line inside each box represents the median; dashed line represents the mean.

The mean WSS values significantly increased at all FP sites following vascular intervention (Figure 4). Median values in the CFA, the SFA, and PA rose from 0.69 (0.56–0.85) Pa, 0.78 (0.70–1.27) Pa, and 0.78 (0.48–0.93) Pa to 0.93 (0.82–1.11) Pa, 1.04 (0.77–1.33) Pa, and 0.91 (0.77–1.22) Pa, respectively.

**Figure 5:**
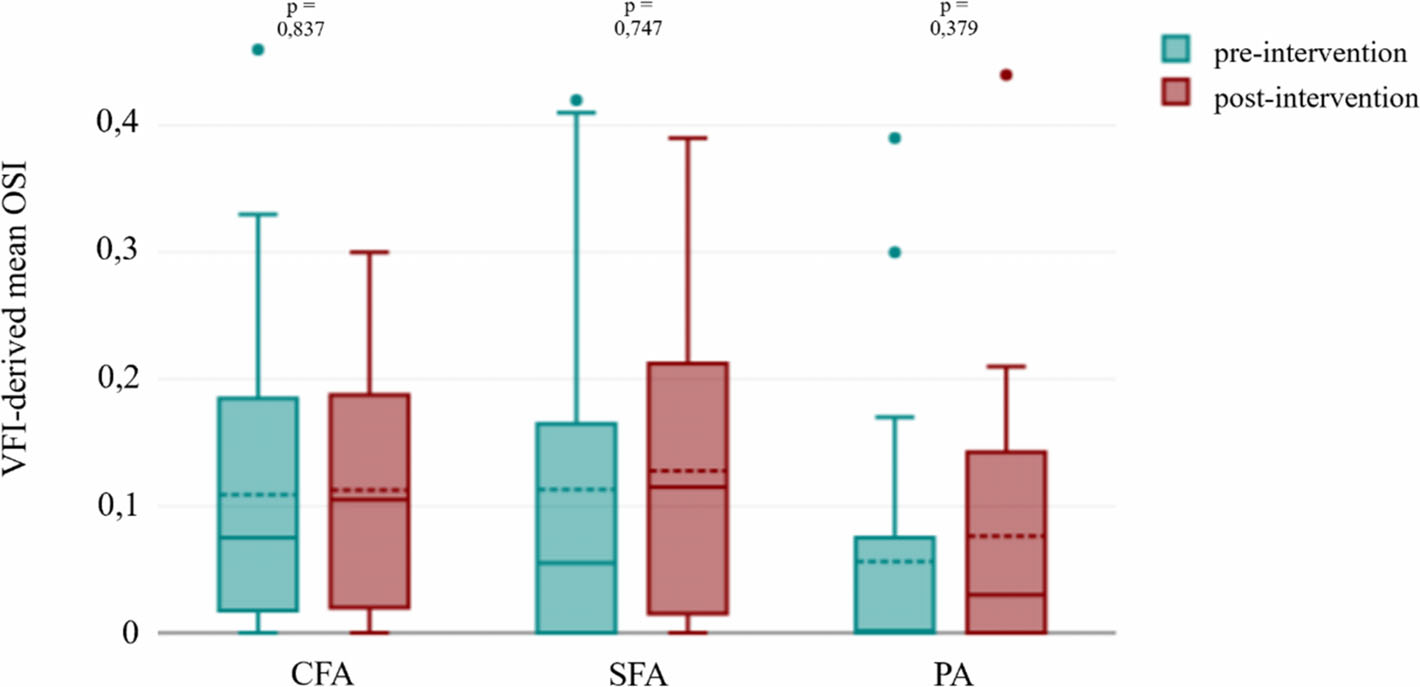
Grouped boxplot of pre‐ and post‐interventional oscillatory shear index (OSI) at 3 femoropopliteal sites. The mean OSI did not significantly change following vascular intervention at CFA, SFA, and PA. CFA, common femoral artery; OSI, oscillatory shear index; PA, popliteal artery; SFA, superficial femoral artery (proximal segment); VFI, vector flow imaging. Solid line inside each box represents the median; dashed line represents the mean.

The mean OSI ranged from a minimum median of 0.0 (pre‐intervention PA) to a maximum of 0.12 (post‐intervention SFA). These changes were not statistically significant (*p* = .837; SFA, *p* = .747; SFA, *p* = .379 for PA; Figure 5).

## Discussion

In this prospective study, we examined hemodynamic parameters before and after iliac or FP artery intervention using conventional Doppler ultrasound and VFI, a high‐frame‐rate ultrasound technique. These interventions resulted in significant hemodynamic changes along the FP axis. Blood volume flow and mean WSS increased at all FP sites, whereas no significant changes in the OSI were observed.

Our study demonstrated that focal iliac or FP revascularization procedures alter hemodynamics, including blood flow‐vessel wall interactions along the FP axis. Using modern ultrasound techniques, the WSS and OSI can be quantitatively assessed during peri‐interventional ultrasound examinations. This allows for further insight into hemodynamic changes that are not currently captured in clinical practice.

At present, the clinical significance of the peri‐interventional increase in WSS observed in our study remains unclear. Under physiological conditions, physiological shear stress prevents atherosclerosis through multiple mechanisms. It stimulates nitric oxide (NO) and adenosine triphosphate production in endothelial cells, promoting the expression of atheroprotective genes. In contrast, low or oscillatory shear stress induces various pro‐atherogenic processes, such as upregulation of pro‐atherogenic genes, increased uptake of low‐density lipoprotein cholesterol, transmigration of leukocytes and vascular smooth muscle cells, and degradation of the extracellular matrix. In the context of vascular interventions, low WSS is associated with postprocedural neointimal formation and restenosis,^7^ which remains a major challenge after revascularization in PAD.^6^ Notably, even in the upright position, the mean shear rate in the SFA is approximately half that of the brachial artery, which may contribute to the “higher propensity for atherosclerosis in the arteries of the leg.”^16^ Specifically, in stented FP lesions, an association has been reported between areas of low WSS and lumen narrowing, whereas no correlation was found between in‐stent restenosis and OSI.^17^ In our study, OSI values did not differ significantly after intervention.

According to recent PAD guidelines,^12^ ultrasonography remains the primary diagnostic modality for detection, intervention planning, and follow‐up. Traditionally, color and PW Doppler imaging is used to assess vascular hemodynamics. In this study, we additionally applied VFI, a plane‐wave imaging technique that enables angle‐independent determination of the magnitude and direction of blood flow velocity vectors at high spatial and temporal resolutions.^13^ Clinically, VFI was initially implemented in the carotid artery, where it showed high inter‐observer reproducibility for WSS measurements.^18^ Our post‐interventional analysis demonstrated a significant increase in volume flow at all examined FP locations, indicating successful revascularization. As previously described, VFI‐derived flow values are typically lower than those derived from PW Doppler due to differences in measurement principles.^14^ Volume flow measurement with pulsed‐wave Doppler assumes a parabolic velocity profile. However, particularly in arteries affected by atherosclerosis, the actual velocity profile can range between parabolic and flat^19^ and may include focal turbulence, introducing a potential systematic error in PW‐derived volume flow. The dispersion of the differences in our study increased with larger mean values, suggesting the presence of proportional bias. Similarly, in a previous study,^20^ volume flow estimations assessed by PW Doppler were found to be overestimated compared to phase contrast MRI estimations, whereas VFI estimations showed a much better correlation. Importantly, the spreads of the VFI measurements were smaller than those of PW versus MRI and no systemic bias for VFI was found. To our knowledge, no prior study has directly compared PW Doppler‐, VFI‐, and MRI‐derived volume flow measurements in the FP artery of patients with PAD. In addition to using MRI as the reference standard, comparison with alternative ultrasound methods may help to assess the accuracy of VFI‐derived volume flow. For example, a 3D method has been reported that is independent of Doppler angle, flow profile, and vessel geometry,^21^ and has been tested under pulsatile conditions in laboratory animals^22^ as well as in patients with a portosystemic shunt.^23^ However, beyond addressing technical challenges, the clinical relevance of peri‐interventional volume flow analysis in patients with PAD remains to be determined.

Only a few studies have evaluated WSS along the FP axis using ultrasound. A previous study using PW‐Doppler to calculate WSS found no decline in WSS along the FP axis.^24^ Interestingly, measurements taken after exercise showed a lower increase in WSS in the Hunter's canal,^24^ a known site predisposed to atherosclerosis. The discrepancy between our findings and those results may be due to differences in patient populations: the previous study examined healthy individuals, while our cohort consisted of patients with advanced PAD. The same group later evaluated patients after SFA stent implantation and observed a decline in WSS along the stented segment,^9^ attributing this mainly to the placement of a relatively “rigid” stent within a compliant artery. However, the local stiffness of the FP arteries in patients with symptomatic PAD and senior‐aged volunteers without PAD is much higher than that in young healthy volunteers.^25^ Overall, the clinical implications of changes in WSS and OSI, such as their effects on atherosclerosis progression or restenosis in FP lesions, remain poorly understood, emphasizing the need for further studies. Since ultrasound is the first‐line imaging modality in clinical practice, improved peri‐interventional hemodynamic assessment with VFI may help identify patients at high risk of re‐stenosis and, consequently, support more individualized and tailored therapy. As shown in this study, ultrasound‐based assessments that are feasible for routine clinical use are already available.

## Limitations

We have to acknowledge that our study had some limitations. First, this was a single‐center study with a relatively small and heterogeneous patient cohort, which may limit generalizability. For example, patients with iliac and FP lesions at varying clinical stages were included. Second, it is important to note that VFI is a relatively novel technique with no established reference standards or validated thresholds. No reference modality (eg, MRI) was included in this study for comparison. Additionally, several technical challenges are associated with VFI in the FP artery in PAD, such as deeper vessel location compared to the carotid artery, heavy calcifications with acoustic shadowing,^11^ and local hematoma after peripheral vascular interventions. Therefore, the clinical use of VFI may be limited in some cases, particularly in patients with 1 or more of the technical challenges mentioned above. Third, the study was not designed to assess long‐term outcomes, as no follow‐up examinations were conducted beyond the post‐interventional period. Further research is necessary to confirm the findings regarding hemodynamic changes observed in this study.

## Conclusions

Revascularization procedures in PAD are associated with alterations in the hemodynamic interactions between blood flow and the vessel wall. The clinical implications of increased WSS along the FP axis warrant further investigation. The use of VFI in peri‐interventional settings provides new opportunities to enhance our understanding of vascular hemodynamics and their potential impact on clinical outcomes.

## Data Availability

The data that support the findings of this study are available from the corresponding author upon reasonable request.
